# Clinical features, surgical treatment, and long-term outcomes of moyamoya disease in a single institution of Fujian, Southeast China: A retrospective study

**DOI:** 10.1097/MD.0000000000035684

**Published:** 2023-10-27

**Authors:** Yi-hang Ding, Jing-yi Chen, En-shuang Zheng, Zi-qing Wang, Ri-sheng Liang, Song-sheng Shi, Xian-kun Tu

**Affiliations:** a, Department of Neurosurgery, Fujian Medical University Union Hospital, Fuzhou, China; b, Department of Radiology, Fujian Medical University Union Hospital, Fuzhou, China.

**Keywords:** China, epidemiology, moyamoya disease, outcome

## Abstract

At present, detailed demographic and clinical data of moyamoya disease (MMD) in the population of Southeast China are lacking. Therefore, this study aimed to evaluate the epidemiological and clinical features of MMD in Southeast China. Our cohort included 170 patients diagnosed with MMD over the preceding 5 years. Clinical characteristics were obtained through a retrospective chart review, while follow-up information and outcomes were obtained through clinical visits and imaging. The median age at symptom onset was 49 years (range 4–73), with a peak in the age distribution observed at 41 to 60 years. The female-to-male ratio was 1.125 (90/80), and the ratio of the ischemic type to the hemorrhagic type was 2.33 (119/50). The most common initial symptom was an ischemic event. The 5-year Kaplan–Meier risk of stroke was 4.9% for all patients treated with surgical revascularization. Of all patients, 83.9% were able to live independently with no significant disability, and 89.8% showed improved cerebral hemodynamics. Our study provided detailed demographic and clinical data on Southeastern Chinese patients with MMD, which was consistent with findings in other parts of China. Raising clinical awareness of MMD in primary hospitals is important to facilitate early diagnosis and timely treatment of MMD patients.

## 1. Introduction

Moyamoya disease (MMD) is characterized by progressive stenosis or occlusion of the intracranial vessels,^[1]^ and has a high prevalence in Asian countries, particularly Japan, South Korea, and China.^[2–4]^ Clinical presentations include ischemic and hemorrhagic stroke and cognitive impairment, which can occur in both children and adults, thus resulting in high incidences of disability and death.

The incidence of MMD worldwide has been well-documented,^[5–12]^ including the incidence in Japan, South Korea, Europe, and the United States, An upward trend was observed. In China, the relatively accurate national epidemiological data reveals that the annual incidence has increased. One study that used the quality monitoring system of a Chinese Hospital reported that the annual incidence of MMD was 1.14 cases per 100,000 inhabitants from 2016 to 2018.^[13]^ Another study using the database of China Urban Basic Medical Insurance revealed a national crude incidence of.59 and prevalence of 1.01 per 100,000 person-years in 2016.^[14]^ China’s land area is approximately 9.6 million square kilometers and China’s territory spans nearly 50° latitude from north to south and more than 60° longitude from east to west; it has 34 provinces, municipalities, and autonomous regions. Therefore, national statistics cannot accurately reflect the situation in different regions in China. The prevalence, clinical features, treatment, and long-term outcomes of MMD in each region have not yet been well documented, apart from the studies conducted in Nanjing^[15]^ and Taiwan.^[16,17]^ The annual incidence of MMD was.43 per 100,000 inhabitants from 2000 to 2007 in Nanjing, and.15 per 100,000 inhabitants from 2000 to 2011 in Taiwan, which were lower than the annual incidence in China as a whole. Differing incidence rates may be related to the year and method of data collection; however, they also point to the need to analyze regions separately.

At present, detailed demographic and clinical data on the population of Southeast China are still lacking. Therefore, this study aimed to evaluate the epidemiological and clinical features of MMD in Southeast China.

## 2. Methods

### 2.1. Patient selection

We identified 170 MMD patients diagnosed using cerebral digital subtraction angiography (DSA) and magnetic resonance angiography at the Fujian Medical University Union Hospital between 2017 and 2022. This study was approved by the ethics board of the Fujian Medical University Union Hospital and the requirement for consent was waived due to the retrospective nature of the study.

### 2.2. Retrospective chart review

Clinical records, including hospital charts and clinical notes, were reviewed. All data were collected between May 2017 and May 2022. The initial presentations were classified into 3 subgroups: ischemia, including transient ischemic attack (TIA) and ischemic stroke; hemorrhage, including subarachnoid, intraparenchymal, or intraventricular hemorrhage; and other symptoms, including headache, seizure, and asymptomatic (patients were incidentally identified during their annual checkup). Treatments included combined bypass surgery, indirect bypass surgery, and conservative management.

Surgery-related infarction was defined as a new, acute cerebral infarction visible on imaging occurring within 2 weeks after surgery. Transient neurological dysfunction (TND) was defined as neurological dysfunction present before discharge with no imaging abnormalities and a complete recovery. In addition, the evaluation of cerebral hemodynamics after cerebral revascularization is clinically meaningful for the assessment of therapeutic effects; therefore, we performed several examinations in the short and long term after surgery, including brain computed tomography (CT) perfusion (CTP), arterial spin labeling (ASL), and CT angiography (CTA).

### 2.3. Surgical procedure

**Figure 1.**
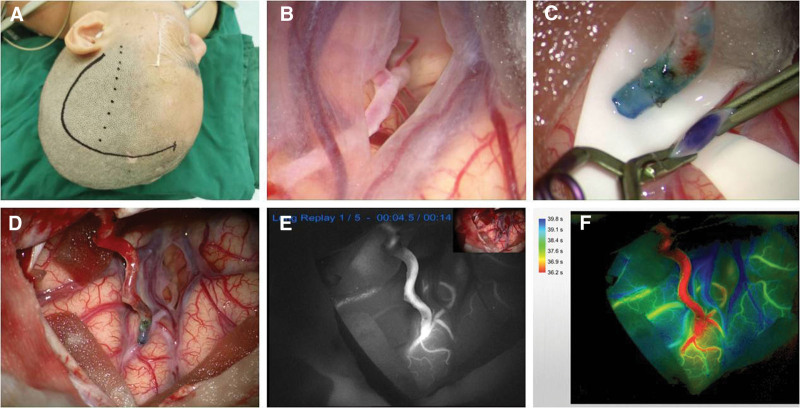
Surgical procedure. A) After anesthesia, the patient was placed in a supine position with the head turned 70° toward the opposite side to the surgery. B) The recipient blood vessels (M4 segment of the middle MCA) are exposed after cutting the arachnoid. C, D) STA was trimmed and anastomosed end-to-side to the M4 segment of the MCA. E, F) Indocyanine green angiography was used to evaluate the bypass patency. MCA = middle cerebral artery, STA = superficial temporal artery.

Most patients in our department underwent combined superficial temporal artery-middle cerebral artery anastomosis and encephaloduroarteriomyosynangiosis. Pediatric and adult patients with severe vascular conditions received encephaloduroarteriomyosynangiosis. All surgeries were performed by an experienced vascular neurosurgeon (X.K.T). After anesthesia, the patient was placed in a supine position with their head turned 70° toward the opposite side of the surgical site. The trunk of the STA and its branches were marked on the skin, and the surgery was performed accordingly. The flap was dissected between the galea aponeurotica and the temporal fascia, and the temporal muscle was completely cut off from the bony surface. The STA was then dissected under a microscope. The dura mater was incised along the middle meningeal artery into strips of width 0.5 to 0.8 cm. The remaining dura was radially incised and turned inward. The recipient blood vessels (the M4 segment of the MCA) were exposed after the arachnoid was cut. The STA was trimmed and anastomosed to the M4 segment of the MCA. Indocyanine green angiography (FLOW 800; Inc., Oberkochen, Germany) was used to evaluate the bypass patency (Fig. 1). The temporal muscle covered the exposed cortex and was anchored to the edge of the bone window. The skull flap was processed to an appropriate shape and fixed without compressing the STA or temporal muscles.

### 2.4. Clinical follow-up

**Figure 2.**
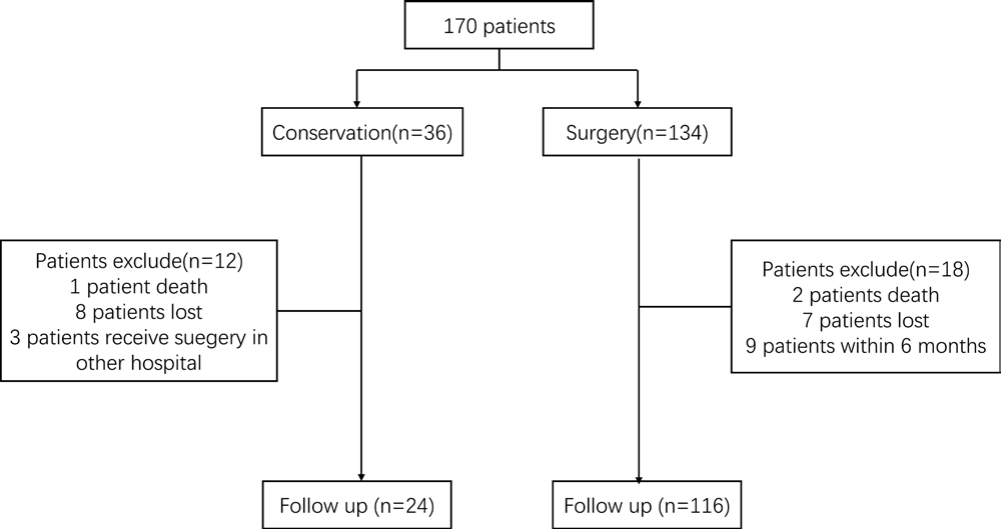
Flowchart of patient follow-up.

After discharge, long-term outcomes were assessed through clinical visits, telephone interviews, or WeChat interviews. In addition, stroke events and modified Rankin scale (mRS) scores were recorded to evaluate patient outcomes. The detailed procedure is illustrated in Fig. 2.

### 2.5. Follow-up imaging

Follow-up imaging included CTP and ASL. CTA was also performed to evaluate the angiographic outcomes of revascularization. All the images were interpreted independently by 2 experienced neuroradiologists.

ASL is a magnetic resonance imaging technique used to assess cerebral perfusion. Assessment of the external carotid artery blood supply in MMD patients using ASL techniques is increasingly common. Semi-quantitative assessment of the perfused area after surgery using external carotid artery (ECA) angiography was graded into 3 levels as follows: poor (the area perfused by the synangiosis is less than one-third of the MCA territory); fair (the area perfused by the synangiosis is between one and two-thirds of the MCA territory); good (the area perfused by the synangiosis is greater than two-thirds of the MCA territory).^[18]^

CTP assesses the changes in the cerebral blood flow (CBF) before and after surgery. Based on the imaging examination results, the development of postoperative cerebral perfusion was semi-quantitatively evaluated and defined at 3 levels: worse, no change, and improved.^[19,20]^ CBF, cerebral blood volume (CBV), time to peak, mean transit time were recorded.

### 2.6. Statistical analysis

SPSS (version 22.0, Armonk, NY) and R (URL: http://www.R-project.org) software were used for data analysis. Continuous variables were expressed as the mean ± standard deviation if the data followed a normal distribution, otherwise the data were presented as the median (interquartile range). Categorical variables were compared using the chi-square test. The Kaplan–Meier method was used to compare the subsequent risk of stroke between the conservative treatment and surgery groups, the adult and child groups, the ischemic and hemorrhagic groups, and the indirect and combined groups. Statistical significance was set at *P <* .05.

## 3. Results

### 3.1. Demographic data

Between May 2017 and May 2022, 170 patients underwent 174 revascularization procedures (Table 1). The results of our study showed a increase in the incidence rate of MMD from 2017 to 2022. In 2017, 15 patients had MMD, and the number increased to 21, 43, 54, and 37 in 2018, 2019, 2020, and 2021, respectively, indicating an increasing trend during this period. However, due to the influence of COVID-19, the number of patients decreased slightly in 2021 and 2022.

**Table 1:** Clinical feature of 170 patients with MMD.

Characteristic	No. of patients (n = 170)
Age, year	49 (range 4–73)
Sex, female: male	90/80
Initial symptoms (no. [%])	
Infraction	68 (40)
Hemorrhage	51 (30)
TIA	35 (20.5)
Headache	8 (4.7)
Asymptomatic	5 (2.9)
Seizure	3 (1.8)
**Suzuki angiographic stage (no. [%]**)	**No. of patients (n = 121**)
2	1 (8)
3	33 (27.3)
4	82 (67.8)
5	5 (4.1)
mRS score at admission (no. [%])	
0–2	128 (75.3)
≥3	42 (24.7)
History of risk factors (no. [%])	
Hypertension	58 (34.1)
Diabetes	22 (12.9)
Hyperthyroidism	7 (4.1)
Smoking or alcohol use	43 (25.3)
Surgical revascularization (no. [%])	174
Combined surgery	110 (63.2)
EDAMS	64 (36.8)
**Year**	**No. of patients**
2017.5–2018.5	15
2018.6–2019.5	21
2019.6–2020.5	43
2020.6–2021.5	56
2021.6–2022.5	37

EDAMS = encephaloduroarteriomyosynangiosis, MMD = moyamoya disease, mRS = modified Rankin scale, TIA = transient ischemic attack.

**Figure 3.**
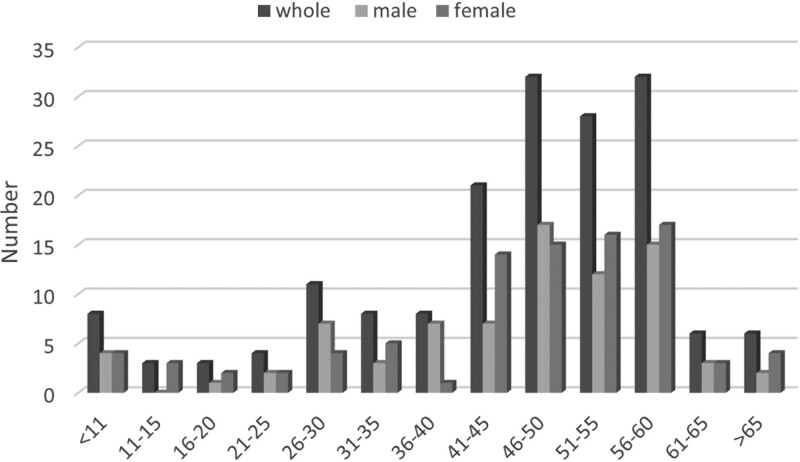
Onset age distribution of moyamoya disease.

The median age at symptom onset was 49 years (range 4–73 years). A peak in the age distribution was observed at 41 to 60 years. The percentages of patients who were ≤ 18 years (n = 12) and those > 18 years (n = 158) were 7.06% and 92.4%, respectively. Both females and males followed the same age distribution pattern (Fig. 3). The female-to-male ratio was 1.13 (90/80).

The ratio of the ischemic type to the hemorrhagic type was 2.33 (119/51), and regarding initial symptoms, 68 patients experienced ischemia, 51 hemorrhage, and 51 other initial symptoms. Of the 170 patients, 36 underwent conservative management, while 134 (78.8%) underwent neurosurgical revascularization, with 40 patients undergoing bilateral revascularization. The median follow-up period after surgery was 24 months (range, 6.0–57.0 months).

### 3.2. Disease type

**Figure 4.**
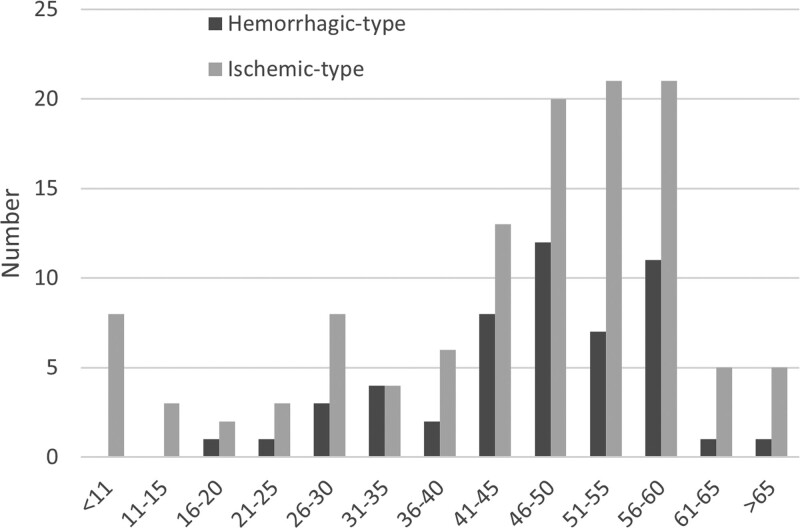
Age distribution of disease pattern at onset.

In our study cohort, infarction was the most common initial clinical manifestation (40.0%). Other initial presentations included hemorrhage (30%), transient ischemic attack (20.5%), headache (4.7%), asymptomatic (2.9%), and seizures (1.8%) (Table 1). The percentage of patients with ischemia as the initial symptom was significantly higher than that of hemorrhage, regardless of age. Hemorrhage, as an initial manifestation, mostly occurred in patients in their fourth and fifth decades of life (Fig. 4). The percentage of patients with hemorrhage as the initial symptom was significantly higher in adult patients than in those ≤ 18 years (*P =* .043) (Table 2). The majority of patients (67.8%) presented with Suzuki angiography stage 4 disease. A higher Suzuki grade was assigned when there was a difference in grades between the 2 sides.

**Table 2:** Clinical presentations (infraction and hemorrhage) of MMD in pediatric and adult patients.

	Age < 18	Age ≥ 18	*P* value
Ischemic (n = 119)	12	107	.043
Hemorrhage (n = 51)	0	51	

MMD = moyamoya disease.

### 3.3. Treatment

A total of 36 patients (19.4%) received conservative management, and 3 later underwent revascularization surgery in other hospitals (3/36, 8.3%). Of the 36 patients, one experienced an ischemic stroke (2.7%) and one experienced a hemorrhagic stroke (2.7%). In addition, one patient died of a heart attack (2.7%).

**Figure 5.**
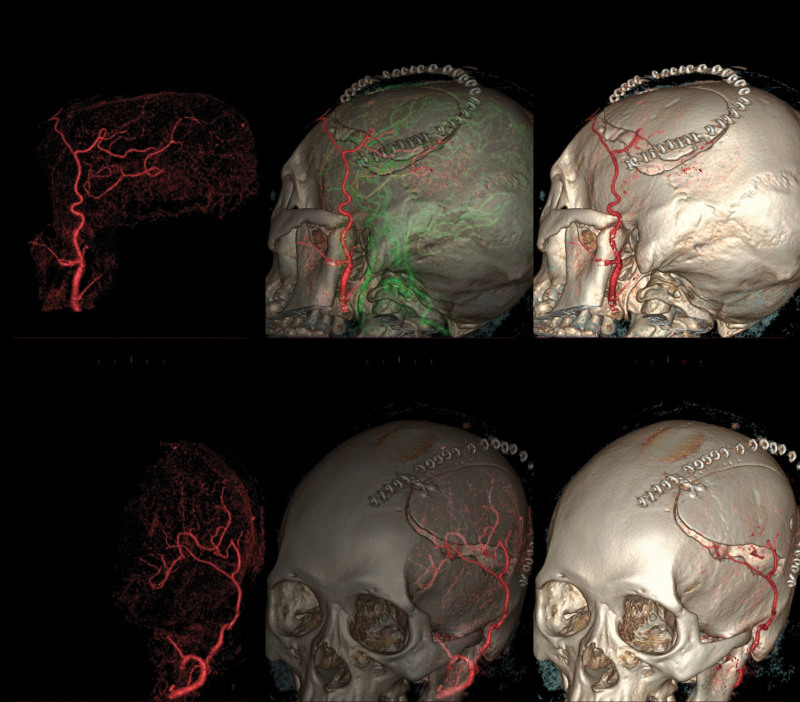
Representative CTA images showing bypass patency in a patient who had undergone left combined bypass surgery. CTA = computed tomography angiography.

The majority of patients, 134 (80.6%), underwent neurosurgical revascularization. Forty patients underwent bilateral procedures; therefore, a total of 174 procedures were performed. Combined bypass surgery (n = 110) was the most common type of procedure performed, with a patency rate of 90% as observed by CTA on postoperative day 7 (Fig. 5). Sixty-four indirect bypass surgeries were performed, and the mean length of hospital stay was 15.24 days. During the postoperative period (defined as the first 30 days after the revascularization procedure), 5 patients (2.9%) experienced ischemic stroke, while none experienced hemorrhagic stroke. One patient died of surgical complications during the postoperative period. We observed TND in 21 hemispheres (12.07%) between days 2 and 14 after the surgery.

### 3.4. Follow-up

The median follow-up period after surgery (n = 134) or conservative management (n = 36) was 22.4 months (range, 5–79 months) (Table 3).

**Table 3:** Long-term outcomes of 140 patients with 6-month minimum follow-up.

Outcomes	Treatment		*P* value	Types of surgical patients		*P* value
	Surgical (n = 116)	Conservative (n = 24)		Ischemia (n = 88)	Hemorrhage (n = 28)	
Follow-up events (no. [%])						
New bleeding	3 (2.6)	1 (4.2)	.53	0	3 (10.7)	
Ischemic event(s)	3 (2.6)	1 (4.2)	.53	3 (3.4)	0	
Functional outcomes (no. [%])						
Improvement in mRS score	66 (56.9)	10 (41.7)	.17	53 (60.2)	13 (46.4)	.20
mRS Score 0–2	104 (89.7)	18 (75.0)	.05	81 (92.0)	23 (82.1)	.13
mRS Score ≥ 3	12 (10.3)	6 (0.25)	.05	7 (8.0)	5 (17.9)	.13

mRS = modified Rankin scale.

**Figure 6.**
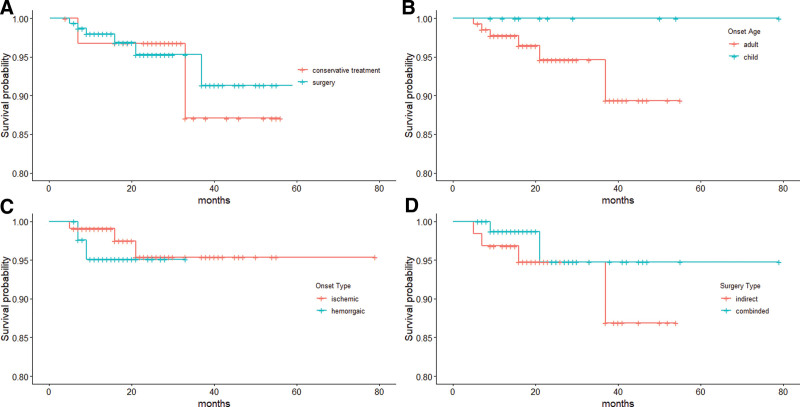
Kaplan–Meier plot for stroke-free survival after surgery for all patients treated with surgical revascularization (A), different age of onset (B), different surgery type (C), and different disease type (D).

Among the 134 surgical patients, 2 died during the postoperative period, 7 could not be contacted for data collection by phone or letter, and 9 were followed-up for less than 6 months after surgery. Therefore, the median follow-up period after surgery (n = 116) was 24 months (range, 1.0–57.0 months). The ischemic group showed a significant improvement in mRS score (Table 4). During the follow-up period 6 strokes (5.2%) occurred in 116 patients, with 4 patients experiencing hemorrhagic stroke and 2 experiencing ischemic stroke. Two ischemic and 3 hemorrhagic strokes occurred within the first 2 years after revascularization surgery and 1 hemorrhagic stroke occurred more than 2 years after surgery. The pediatric patients had no recurrent stroke during the follow-up period. The Kaplan–Meier estimate for recurrent stroke was 2.3%. The 5-year-Kaplan–Meier risk of recurrent stroke was 4.9% after surgery in patients who had undergone surgical revascularization. The analyses of childhood- and adult-onset MMD, different surgery types, and disease types were done separately (Fig. 6).

**Table 4:** Postoperative mRS changes of different types of moyamoya disease.

mRS score	Pre-operation	Post-operation	*P* value
Hemorrhagic type (n = 38)			
0	12	17	.122
1	6	9	
2	11	6	
3	5	4	
4	3	0	
5	1	2	
Ischemic type (n = 88)			
0	10	47	<.01
1	69	45	
2	12	7	
3	12	5	
4	4	2	
5	0	1	

mRS = modified Rankin scale.

### 3.5. Follow-up evaluation of cerebral hemodynamics

**Figure 7.**
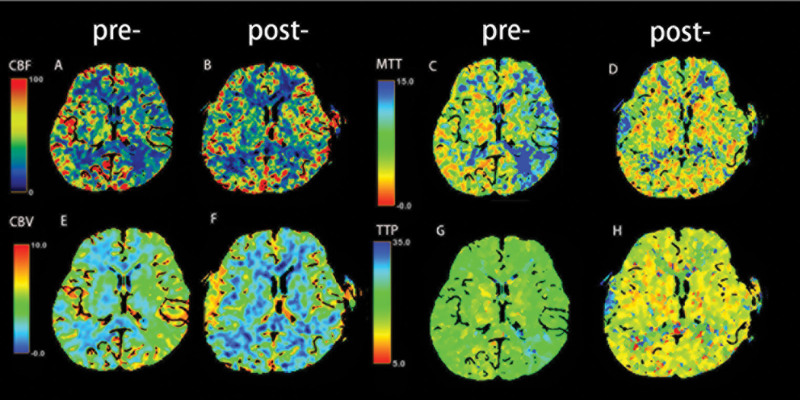
CT perfusion images before and after surgery. Images show cerebral hemodynamics changes pre and postoperatively of a right-sided superficial temporal artery-to-middle cerebral artery anastomosis in moyamoya disease. Axial perfusion CT images show reduced (A) CBF, delayed (C) MTT, increased (E) CBV, and (G) TTP in the region of right middle cerebral artery before surgery. The postoperative CTP show increased (B) CBF, reduced (D) MTT, (F) CBV and (H) TTP in the region of right middle cerebral artery. CT = computed tomography, CTA = computed tomography angiography, CTP = computed tomography perfusion, MTT = mean transit time, TTP = time to peak.

A total of 145 cerebral hemispheres were examined through imaging in 116 patients, including 81 who had undergone combined bypass surgery and 64 who had undergone indirect bypass surgery. The interval between surgery and follow-up imaging was at least 6 months, and patients who received follow-up imaging within 6 months of surgery were excluded. CTP and ASL were performed in 108 MMD patients (Table 5). The CTP result showed improved cerebral hemodynamics in the affected hemispheres of 97 patients (89.8%), as presented in Fig. 7. Compared to the preoperative values, the postoperative CTP result showed an increased CBF (B) and reduced mean transit time (D), CBV (F), and time to peak (H) in the region of the right MCA.

**Table 5:** Postoperative imaging of different types of surgery.

	Combined bypass (n = 70)	Indirect bypass (n = 38)	*P* value
CTP			
Worse (no. [%])	3 (4.3)	1 (2.6)	.882
No change (no. [%])	4 (5.7)	3 (7.9)	
Improve (no. [%])	63 (90)	34 (89.5)	
ASL			
Poor (no. [%])	4 (5.7)	2 (5.3)	.458
Fair (no. [%])	6 (8.6)	6 (15.8)	
Good (no. [%])	60 (85.7)	30 (78.9)	

ASL = arterial spin labeling, CTP = computed tomography perfusion.

**Figure 8.**
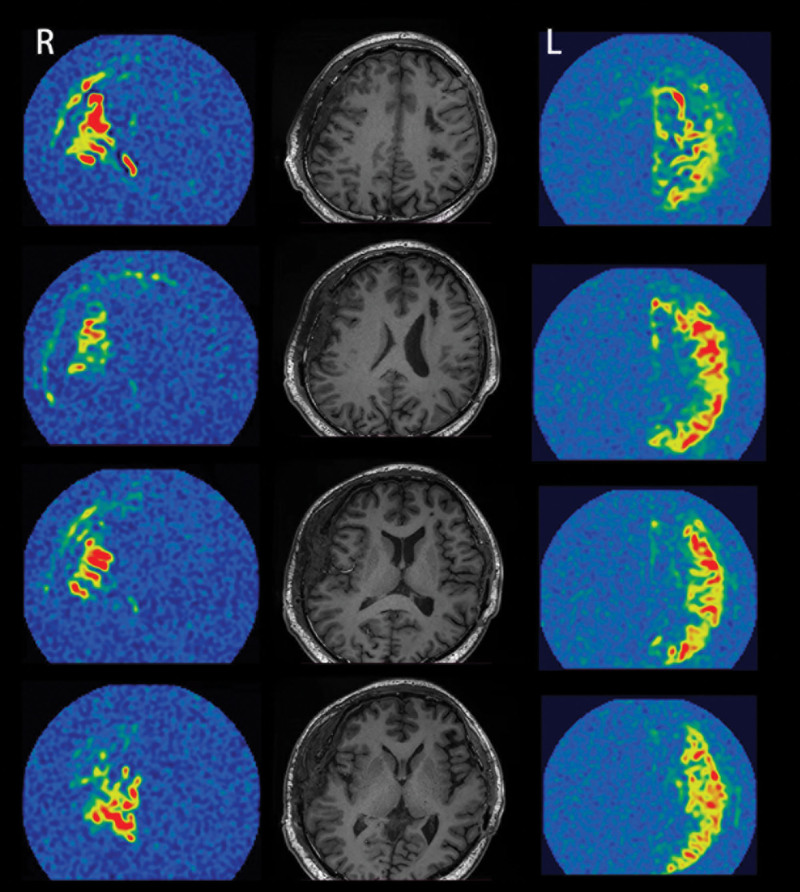
Representative ASL images showing good postoperative perfusion of the external carotid artery in a patient who had undergone left combined bypass surgery 6 months prior and right combined bypass surgery 1 week prior. ASL = arterial spin labeling.

Regarding ASL, CBF changes were classified as grade 1 in 6 patients (5.5%), grade 2 in 12 patients (11.1%), and grade 3 in the remaining 90 patients (83.3%) (Fig. 8).

## 4. Discussion

MMD is a chronic cerebrovascular disorder mainly found in Asia, especially in Japan, South Korea, and China.^[1]^ However, given China’s vast territory, several socioeconomic and environmental factors may play an important role in the regional differences in the clinical features of MMD within China.^[14]^ Although MMD has been reported in other parts of China,^[13,14,21]^ the epidemiological features of MMD in Southeast China remain unknown. Upon reviewing the literature, one realizes that there is minimal knowledge regarding the clinical features, surgical treatment, and long-term outcomes of MMD in Southeast China. Therefore, we present data from a cohort of 170 MMD patients in Fujian, Southeast China, with details of the clinical manifestations and outcomes. Although our hospital cannot reflect the incidence rate of the entire Fujian province, our center is one of the most important medical institutions in Fujian province and receives the majority of MMD patients. Notably, there were several differences between the MMD patients in our cohort and patients from other countries. However, our data are similar to a nationwide Chinese cohort study.

MMD is currently attracting increasing attention in Southeast China, as reflected by the increasing incidence rates. The low awareness of MMD in the medical profession and society may partly explain its low incidence in the early years. In line with our observations, previous investigations, such as those in Japan and Korea, have shown an increased incidence of MMD over time.^[3–5]^ These increases in the incidence of MMD are probably due to greater detection rather than an epidemiological increase. With advancements in knowledge regarding MMD and neuroradiological technology, including MRA and CTA, more patients who were previously missed can now be diagnosed. MRA and CTA can show the stenosis of the internal carotid artery and moyamoya vessel. DSA is the gold standard, but in most cases MRA and CTA are of great help in diagnosing MMD. In addition, improving the knowledge related to MMD in primary hospitals is important because many MMD patients with cerebral infarction or hemorrhage visit primary hospitals first. Especially in patients with recurrent TIAs, if MMD can be diagnosed earlier, serious sequalae can be avoided.

In our cohort, the ratio of females to males was 1.125, which is similar to reports from Beijing,^[21]^ Taiwan,^[16]^ Nanjing,^[15]^ and China as a whole.^[13,14]^ The results suggest no significant difference exists in the sex distribution of MMD in China.^[14]^ However, reports from Japan, South Korea, the United States, and Europe showed a female predominance.^[2,22]^ This may be due to genetic differences between China, Japan, South Korea, and other places, and the underlying mechanism remains to be analyzed.

The typical peak childhood age for MMD in Asian patients was not observed in our study. The number of children was small, and the results differed from those in Japan, South Korea, and other parts of China.^[15,21]^ We believe that the current data from our center for children with MMD do not reflect the true incidence, so there is no report on the incidence of pediatric MMD. The reason for the low percentage of children is unknown. We ascribe this phenomenon simply to an insufficient understanding of the disease among clinicians. Many pediatricians have limited knowledge about MMD, making it difficult to diagnose cases accurately and promptly without specific examinations. This could result in misdiagnosis and inadequate treatment of pediatric patients.

The peak incidence of MMD was observed in patients aged 41 to 60 years. MMD is a progressive disease; therefore, patients often visit our hospital after serious events, such as cerebral infarction or hemorrhage. Prior to this, they may have ignored significant symptoms such as a TIA. As the number of newly diagnosed patients increased, the proportion of older patients also increased. This is similar to reports from other studies in China, suggesting an adult predominance.^[13,14]^

In our cohort, the patients present with a ratio of ischemic to hemorrhage of 2.3, which differed from findings in Japan and Europe.^[2]^ The reason for the high percentage of the ischemic type may involve various factors. One explanation is that ischemic symptoms develop soon after the onset of arterial narrowing or occlusion.^[23]^ Another explanation is the routine use of CTA or MRA in patients with cerebral infarction. In contrast, ischemia patients may not know they suffer from MMD. The JAM Trial from Japan revealed that direct bypass surgery for adult patients with hemorrhagic MMD reduces the rebleeding rate and improves patient prognosis.^[24]^ Many mainstream doctors have not changed their view that surgical treatment of hemorrhagic MMD is effective. Therefore, they may not recommend surgical treatment to those patients, excluding some potential patients. Recently, with advances in detection technology for MMD, more patients with the hemorrhagic type, who were previously missed, can now be diagnosed, which is reflected by the increased incidence of the hemorrhagic type.

The percentage of asymptomatic MMD patients was low in our study. Most of these patients are incidentally identified using DSA or MRA. However, due to economic limitations, routine DSA and MRA checkups are not feasible in large populations. Therefore, more accurate detection and improvements in the medical system and financial capabilities of the patients in China are required.^[21]^ Radiological methods, such as CTP, CTA, and ASL, have been used to assess hemodynamics in MMD, which can help identify patients with hemodynamic cerebral insufficiency and assess hemodynamic improvement after revascularization surgery.^[25]^

In previous studies, TND was the most common perioperative complication.^[26]^ In our study, the incidence of TND was 12.07%, which was comparable to that in most studies, where the incidence rate was reported to be 20% to 60%.^[26]^ Surgery-related infarction was also observed in the study and cases were closely associated with unfavorable functional outcomes. Based on our observations, the occurrence of TND may be associated with postoperative hyperperfusion syndrome and watershed syndrome.^[26,27]^ For patients with postoperative cerebral infarction, frequent preoperative TIAs, which indicate an insufficient blood supply to the brain, may be a risk factor and further prospective studies are needed. Careful attention should be paid to maintaining an adequate hemodynamic status during the perioperative period.^[23,27,28]^

The main goal of revascularization surgery in MMD patients is to prevent future ischemic and hemorrhagic strokes and potentially limit disease progression. In this study, most patients had good outcomes during the follow-up period. Regarding clinical data, a trend for better outcomes in surgically treated patients is observed, and the incidence of postoperative stroke was 5.2%, which is comparable to most other large studies, with postoperative complications between 3.5% and 7.7%.^[23,29,30]^ The 5-year-Kaplan–Meier risk of recurrent stroke was 4.9% after surgery for patients with surgical revascularization. Comparing the Kaplan–Meier risk of different types of patients was challenging because of this study’s small sample size and selection bias. Randomized clinical trials are required to investigate the efficacy of revascularization surgery.

Furthermore, our study confirmed that the mRS score improved in the surgical treatment group compared to that in the conservative treatment group (Table 3, *P* = .05). Additionally, our study confirmed the role of surgical revascularization in treating patients with ischemic MMD (Table 4, *P* < .01). For ischemic MMD, superficial temporal artery-middle cerebral artery bypass flow can improve cerebral perfusion. As for the hemorrhagic type, the cause of hemorrhage is often the rupture of moyamoya vessels or an aneurysm. Although diminishment of moyamoya vessels and aneurysms can be observed after surgery,^[24,31–33]^ a longer follow-up time may be required to establish this fact. In our study, the median follow-up duration was 22.4 months (range, 5–79 months); therefore, a longer follow-up period is required. Regarding imaging data, this study demonstrated an improvement in perfusion after revascularization surgery. CTP and ASL revealed improved perfusion in 89.8% of the patients. Examinations showed that the majority of revascularization surgeries (83.3%) could provide sufficient ECA system blood flow to the MCA territory, indicating that surgery can improve long-term outcomes by improving cerebral perfusion.

This study has several limitations. First, the sample size was small and the follow-up duration was only 5 years. Further follow-up is needed to evaluate the long-term outcomes of revascularization surgery in MMD patients. Second, although our center receives the majority of patients in Fujian, it is a single center study, and multicenter studies will be conducted in the future improve the generalizability of the results. Third, follow-up imaging data were not sufficient, which will also be addressed in the follow-up study. Many other provinces in China, like Fujian province, are just beginning to recognize MMD. As a rare disease, there is still much to do to improve recognition and medical treatment of MMD.

## 5. Conclusion

In conclusion, this study contributes to the current knowledge base on MMD in Southeast China. With the increasing awareness of MMD, and advancements in knowledge and prognosis following treatment at our center, MMD has attracted increasing attention in this region.

## Author contributions

**Supervision:** Ri-sheng Liang, Song-sheng Shi, Xian-kun Tu.

**Validation:** En-shuang Zheng, Xian-kun Tu.

**Writing – original draft:** Yi-hang Ding, Jing-yi Chen.

**Writing – review & editing:** Zi-qing Wang.
